# Protective Effect of Edaravone Against Oxidative Stress in C2C12 Myoblast and Impairment of Skeletal Muscle Regeneration Exposed to Ischemic Injury in Ob/ob Mice

**DOI:** 10.3389/fphys.2019.01596

**Published:** 2020-01-15

**Authors:** Takuya Nakanishi, Masaya Tsujii, Takahiro Asano, Takahiro Iino, Akihiro Sudo

**Affiliations:** Department of Orthopaedic Surgery, Graduate School of Medicine, Mie University, Tsu, Japan

**Keywords:** skeletal muscle, obesity, oxidative stress, myoblast, free radical scavenger, edaravone

## Abstract

**Background:**

The aims of this study were to analyze the effects of the administration of edaravone on C2C12 myoblasts exposed to oxidative stress; to evaluate the skeletal muscles in ob/ob mice; and to analyze the effect of the administration of edaravone in the regeneration of skeletal muscle after ischemic injury.

**Methods:**

In C2C12 myoblasts, oxidative stress was induced by the exposure to 250 μM H_2_O_2_ for 4 h with or without pretreatment of 100 μM edaravone. Thereafter, the viability and expression of TNF-α were analyzed by MTS assay and PCR, respectively. Furthermore, an *in vivo* study was performed on male C57/BL6-ob/ob mice (10 weeks old) and the respective control mice. The skeletal muscles of tibialis anterior and gastrocnemius were excised for histological analysis and TBARS assay after the measurement of blood flow. In addition, the regeneration of the skeletal muscles was analyzed for the expression of MyoD 7 days after the ligation of the right femoral artery.

**Results:**

Edaravone significantly inhibited the reduction of the viability as well as upregulation of TNF-α expression by treatment with H_2_O_2_. In ob/ob mice, wet weight of muscles was significantly lower than that in control mice. In histology, ob/ob mice had significantly less multi-angle shaped myofibers and a significantly high level of MDA. Furthermore, MyoD expression was lower in ob/ob mice than in control mice after the ischemic injury, while edaravone (3 mg/kg) increasingly enhanced MyoD expression.

**Conclusion:**

Edaravone attenuated the oxidative stress on C2C12 myoblasts, and was effective to regeneration of skeletal muscles after ischemia in ob/ob mice.

## Introduction

Dysfunction associated with a decrease in skeletal muscle mass is a major cause of poor quality of life, especially among the elderly (Newman et al., 2006; Enoki et al., 2007; Kohara, 2014). The reduction in skeletal muscle mass and muscle weakness associated with aging is called sarcopenia (Cruz-Jentoft et al., 2010). Although the molecular mechanism of sarcopenia has not been elucidated, several factors were reported to be involved in its pathogenesis, including oxidative stresses, chronic inflammation, and sex hormones (Kohara, 2014).

Oxidative stress is caused by disturbance of homeostasis of ROS, and involves many pathological conditions, such as aging, blood flow disorders, obesity, inflammation, and malignant tumors (Toufektsian et al., 2001; Vincent et al., 2007; Gadjeva et al., 2008; Yang et al., 2013; Conti et al., 2015). The oxidative stress also leads to destructive and irreversible damage in skeletal muscle cells (Tidball, 2011; Choi et al., 2016; Powers et al., 2016). Therefore, it is reasonable that administration of antioxidants could be considered as a strategy for protection from various disorders that affect skeletal muscle. In fact, there exist a number of studies concerning the use of antioxidants in pathological conditions that affect skeletal muscles. H_2_O_2_ has been widely used for induction of the oxidative stress in *in vitro* studies, because previous studies showed that H_2_O_2_ was sufficient and essential to induce oxidative stress on C2C12 mice skeletal myoblasts (Scherz-Shouval and Elazar, 2007; Li et al., 2014). As result, various antioxidants, including adiponectin and interleukin-15, were shown to have protective effects against H_2_O_2_-mediated oxidative stress in C2C12 mice skeletal myoblasts (Li et al., 2014; Ren et al., 2017). Clinical use of antioxidants has been expected to improve atrophy of the skeletal muscles due to oxidative stress, although clinically effective treatment has not been established.

Furthermore, the atrophy of the skeletal muscles could be associated with the obesity. In obese individuals, excessive lipid accumulation in adipose and non-adipose tissues including the skeletal muscle, and subsequent oxidative stress reactions and low-grade inflammation, impair skeletal muscles function due to lipotoxicity (Akhmedov and Berdeaux, 2013). Thus, obesity has an underlying pathophysiology similar to that of sarcopenia in skeletal muscle. Sarcopenia and obesity are considered to have various overlapping etiologies and feedback mechanisms supposed to be strongly interconnected and aggravating each other mutually (Kalinkovich and Livshits, 2017). The comparative study also demonstrated that obese adults had impairment of quadriceps muscle strength compared to non-obese adults (Maffiuletti et al., 2007). Therefore, the combination of both the conditions is called as sarcopenic obesity, which is not just a combination. In fact, sarcopenic obesity could increase the risk of metabolic and physical impairment more than do sarcopenia or obesity alone (Kalinkovich and Livshits, 2017). However, further investigation is required to determine how both the conditions interplay reciprocally.

Leptin, which is considered as a key molecule in the pathophysiology of obesity, could play an important role in sarcopenic obesity, since leptin resistance was observed in subjects with sarcopenic obesity in comparison with subjects having either obesity or sarcopenia (Kohara et al., 2011). In addition, leptin-deficient (ob/ob) mice became insulin-resistant due to deficiency of leptin, and developed severe obesity. These animals have been used in several research studies on the pathophysiology of diseases related to obesity including cardiovascular disease, renal disorder, and diabetic mellitus. Ob/ob mice are likely to be a potent model to elucidate the pathophysiology of sarcopenic obesity on the skeletal muscle. Furthermore, we focused on the reduction of blood flow underlying the atrophy of skeletal muscle in obesity, because individuals with obesity have a significantly increased risk of developing arterial disease, including ischemic heart disease and peripheral artery disease of the lower extremities (Isomaa et al., 2001; Plummer and Hasty, 2008).

The aims of this study were (1) to analyze the effects of oxidative stress and administration of edaravone, which is a synthetic scavenger of free radicals with protective effect against ischemic injuries, on skeletal muscle cells using C2C12 mouse myoblasts, (2) to evaluate the characteristics of the skeletal muscles in ob/ob mice, and (3) to analyze the regeneration of skeletal muscles following ischemic injury and the effect of edaravone in such situation.

## Materials and Methods

### Subjects

*In vitro* study was performed using C2C12 cell lines (ATCC, Manassas, VA, United States). C2C12 mouse myoblasts were cultured in a growth medium consisting of low-glucose DMEM containing 10% FBS in a 5% CO_2_ incubator at the temperature of 37°C. After culturing until reaching 70–80% confluence, C2C12 cells were used for the following analysis.

*In vivo* study was performed on 30 male C57/BL6-ob/ob mice and the same number of their control strain, C57/BL6 mice (10 weeks old; SLC, Hamamatsu, Japan). The animals were housed in a temperature-controlled environment and maintained on a 12-h light–dark cycle with food and water available *ad libitum*. The experimental protocol was approved by the committee of animal research at Mie University.

### Antioxidant Effect to Oxidative Stress on C2C12 Myoblasts

Oxidative stress was induced by exposure to 250 μM H_2_O_2_ for 4 h, which is one of the most widely used ROS to induce oxidative stress in cellular models (Li et al., 2014). Cells were also pretreated with or without 100 μM edaravone for 1 h. Edaravone was purchased from MedChemExpress LLC (South Brunswick Township, NJ, United States). It was dissolved in 1N NaOH that was titrated to pH 7.4 with 1N HCl to prepare a final concentration of 0.3 mg/ml. Thereafter, the viability of C2C12 myoblasts and expression of TNF-α were analyzed by MTS assay and real-time PCR, respectively.

### MTS Assay

Cells were seeded at a density of 15000 cells/well onto a 96-well plate in a 100 μl medium and incubated at 37°C for 24 h. Cells were subjected to oxidative stress as above-mentioned, and proliferation was analyzed according to the manufacturer’s instructions (Promega Corporation, Madison, WI, United States). Twenty microliters of MTS reagent was added directly into the wells and incubated for 2 h. Absorbance was measured at 492 nm on a Multiscan JX microplate reader (Thermo Labsystems, Waltham, MA, United States). The results are represented as the percentage of relative fluorescence units compared to the control without stimulation by H_2_O_2_.

### Quantitative Real-Time PCR

C2C12 cells were harvested and total RNA was extracted from each sample using ISOGEN (Nippon Gene, Tokyo, Japan), according to the manufacturer’s instructions. The RNA was then reverse-transcribed into cDNA using the First Strand cDNA Synthesis kit (Roche Applied Science, Mannheim, Germany). The TaqMan^®^ Gene Expression Master Mix and the TaqMan Gene Expression Assay (Applied Biosystems, Foster City, CA, United States) were used to quantitatively analyze the expression of genes, including those coding for GAPDH and TNF-alpha. Real-time quantitative PCR amplifications were performed using an ABI PRISM^®^ 7000 Sequence Detection System (Applied Biosystems). GAPDH was used as an endogenous housekeeping gene for normalization.

### Experimental Model in *in vivo* Study

Mice were deeply anesthetized with an intraperitoneal injection of sodium pentobarbital (0.05 mg/g body weight) following the measurement of body weight. Then, muscles of TA and gastrocnemius were exposed and measured for blood flow using a laser doppler flowmetry (Nexis Corporation, Fukuoka, Japan). Thereafter, the muscles were fully excised from the attachment site of the bone, and wet weights were measured.

### Histological Analysis

The specimens were transversely cut at 5 μm on a microtome and stained with hematoxylin and eosin (HE). The morphology of each myofiber of the gastrocnemius was observed in all the 15 photograph field on each specimen under the microscope (BX50, Olympus, Japan) (McCormack et al., 2008). In uninjured skeletal muscles, we counted the number of the small myofibers with loss of multi-angle shape on the specimens harvested from control and ob/ob mice. Furthermore, the absolute injury scores of skeletal muscles due to ischemia were determined by a method of McCormack et al. (2008). The muscle injury score was expressed as a percentage, obtained by dividing the number of injured myofibers by the total number of myocytes scored within all the fields. The intraobserver and interobserver reliability correlation coefficient of muscle injuries at two times were excellent (κ = 0.96, 0.91, respectively), as determined by the Cohen kappa correlation coefficient.

### Measurement of Malondialdehyde (MDA)

The lipid peroxidation was assessed by measuring the MDA content of tissue using a TBA assay kit (Northwest Life Science Specialties LLC, Vancouver, WA, United States). The muscle harvested from gastrocnemius (*n* = 5 in both obese and control mice) was immediately frozen in liquid nitrogen after washing with 0.9% NaCl. These resected muscles were then homogenized using Cryopress (Microtech, Chiba, Japan) and stirred in an assay buffer (phosphate buffer, pH 7.0 with EDTA) for 1 h. Butanol was then added to the sample to remove hemoglobin, because malondialdehyde (MDA) or MDA-like substances and TBA can react, producing a pink pigment in the TBA test reaction (Hori et al., 2013). The precipitate was centrifugally pelleted (3 min at 10,000 *g*), and an aliquot of the supernatant was made to react with an equal volume of TBA at 60°C for 60 min. After cooling, sample absorbance was measured at 540 nm. The results for tissue samples are expressed in nmol/mg protein, using the standard graphic prepared according to the measurements performed with a standard solution.

### Surgical Procedure of Ischemic Injury

Under deep anesthesia, the right femoral artery was exposed and ligated at a level between epigastric artery and bifurcation of saphena and popliteal artery (Crawford et al., 2013). Furthermore, 3 mg/kg edaravone or saline solution was injected intraperitoneally 30 min before the femoral artery ligation. As mentioned above, the muscles were excised following measurement of blood flow 7 days after femoral artery ligation (*n* = 9 in both obese and control mice).

### Immunohistochemical Analysis

MyoD expression for proliferating myosatellite cells was examined using tissues of the gastrocnemius muscles harvested from control and ob/ob mice 7 days after ligation of the femoral artery. After paraffin was removed, endogenous peroxidase was inactivated by 0.3% hydrogen peroxide in methanol for 30 min. The sections were incubated with rabbit polyclonal anti-MyoD antibody (Santa Cruz Biotechnology, Santa Cruz, CA, United States) overnight at room temperature. Between the incubation steps, sections were dip immersion washed (3 min × 5-min wash) in TBS to eliminate excesses of non-bound antibody or reagent. The antibody was diluted in 1% BSA/TBS to suppress non-specific reactions. Then, the sections were incubated with the reagent which anti-rabbit immunoglobulin conjugate HRP and anti-mouse immunoglobulin conjugate HRP mixed in by employing the universal immuno-enzyme polymer method (Histofine^®^ Simple Stain MAX-PO; Nichirei, Tokyo, Japan). The reaction products were visualized in 0.15 mg/ml DAB solution containing 0.003% hydrogen peroxide. After washing in water, the counter was stained by hematoxylin.

### Western Blotting

Expression of MyoD was analyzed by western blotting. The muscle tissue harvested from gastrocnemius 7 days after artery ligation (*n* = 5 in each group) was frozen in liquid nitrogen, homogenized using a Cryopress (Microtech, Chiba, Japan) and stirred in the RIPA buffer (10 mM Tris-HCl (PH 7.4), 1% NP40, 0.1% sodium deoxycholate, 0.1% SDS, 0.15M NaCl, 1 mM EDTA, 10 μg/ml aprotinin) for 1 h. The supernatants were separated by SDS-PAGE, transferred to nitrocellulose membranes, and immunoblotted with primary antibodies. The primary antibody was a rabbit polyclonal anti-MyoD antibody (Santa Cruz Biotechnology, Santa Cruz, CA, United States). The bands were visualized using the ECL Western blotting detection system (GE Healthcare Unided Kingdom, Ltd., Buckinghamshire, United Kingdom) and were detected by a LAS-4000 imager (Fujifilm, Tokyo, Japan). Subsequently, the bands were analyzed by ImageJ software (National Institutes of Health, Bethesda, MD, United Stated) to quantify the expression of MyoD. MyoD levels were normalized to beta actin levels for the same animal to control for the possibility of unequal protein loading, and results were calculated as a ratio in relation to the control group.

### Statistical Analysis

Analyses were performed with SPSS version 22.0 statistical package (SPSS, Inc., Chicago, IL, United States). Data were analyzed using the Mann–Whitney *U*-test. *p* < 0.05 was considered statistically significant.

## Results

### Edaravone Could Effectively Protect C2C12 Mouse Myoblasts Exposed to Oxidative Stress Using H_2_O_2_

**FIGURE 1:**
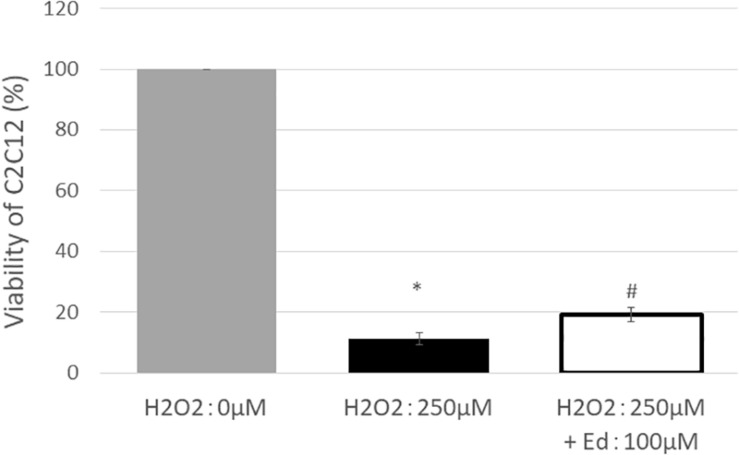
After treating C2C12 cells with 250 μM H_2_O_2_, viability was significantly decreased, and improvement in survival rate was observed in the group treated with edaravone at 100 μM (^∗^compared with 0 μM H_2_O_2_, *p* < 0.01; # compared with 250 μM H_2_O_2_, *p* < 0.01).

**FIGURE 2:**
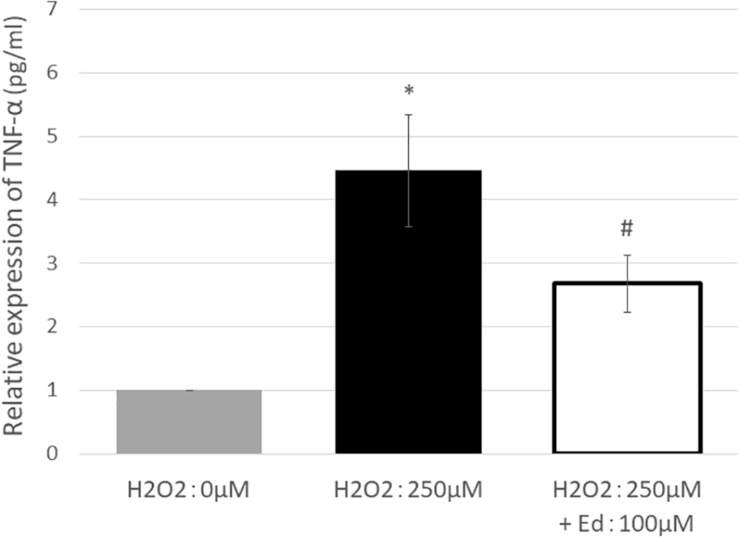
TNF-α expression was significantly increased by treating C2C12 cells with 250 μM H_2_O_2_. In addition, pretreatment with edaravone at 100 μM, TNF-α expression was significantly suppressed (^∗^ compared with 0 μM H_2_O_2_, *p* < 0.01; # compared with 250 μM H_2_O_2_, *p* < 0.01).

Figure 1 shows the viability of C2C12 myoblasts following oxidative stress due to the exposure to H_2_O_2_ by an MTS assay (*n* = 8 in each group). The viability of C2C12 myoblasts was significantly reduced to 11.3 ± 2.20% of the control group by treatment with 250 μM H_2_O_2_, whereas pretreatment with 100 μM edaravone significantly inhibited the reduction in cell viability (18.9 ± 1.53% of the control group), regardless of stimulation by H_2_O_2_. H_2_O_2_ mediated-oxidative stress also significantly upregulated the expression of TNF-α (4.46 ± 0.89 pg/ml) in the C2C12 mice myoblasts compared to the control (1.00 ± 0.80 pg/ml, Figure 2). In addition, the application with edaravone significantly inhibited the upregulation of the expression of TNF-α (2.68 ± 0.45 pg/ml, Figure 2).

### Skeletal Muscles in Ob/ob Mice Had a Significantly Decreased Wet Weight and Had Less Multi-Angle Shaped Myofibers Compared to Those in Control Mice

**FIGURE 3:**
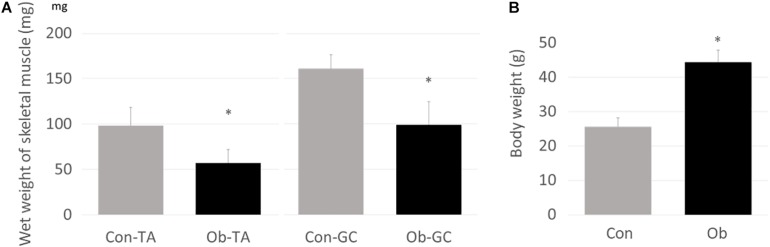
**(A)** Wet weights of both TA and gastrocnemius (GC) muscles were significantly lower in ob/ob mice (Ob) than control mice (Con). **(B)** On the other hand, body weight of Ob was significantly higher than that of Con (^∗^ compared with control mice, *p* < 0.01).

**FIGURE 4:**
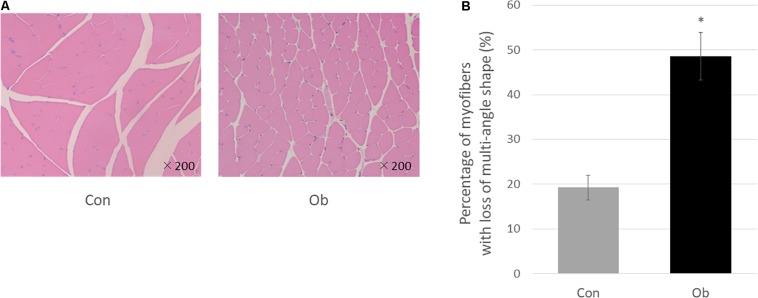
**(A)** HE staining. In control mice (Con), the muscle fibers show multi-angle shape and are in contact with each other, but in ob/ob mouse (Ob), the cells have less multi-angle-shaped fibers. **(B)** Less multi-angle shaped fibers were counted, and in proportion, it was significantly higher in Ob (^∗^ compared with control mice, *p* < 0.01).

**FIGURE 5:**
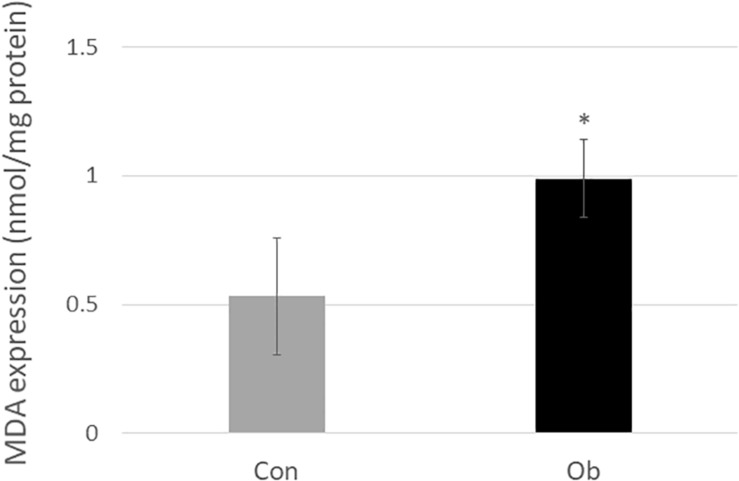
MDA level in the muscle tissue was significantly elevated in ob/ob mice than in control mice (^∗^ compared with control mice, *p* < 0.05).

Wet weights of both TA and gastrocnemius muscles in ob/ob mice were significantly lower than those in control mice (Figure 3A), whereas body weight of ob/ob mice was significantly higher than that of control mice (Figure 3B). Furthermore, ob/ob mice appeared to have a number of small myofibers without loss of multi-angle shape (Figure 4A), whereas myofibers in the skeletal muscles of control mice had a normal architecture with multi-angle shape. In fact, morphologic analysis showed that there existed significantly more myofibers without loss of multi-angle shape in ob/ob mice (48.5%) than in control mice (19.2%, Figure 4B). Furthermore, the extent of lipid peroxidation was determined by MDA analysis in the muscles, with significantly higher levels in ob/ob mice (0.99 ± 0.15 nmol/mg protein) than in control mice (0.53 ± 0.23 nmol/mg protein, Figure 5). Moreover, there were no differences in blood flow to skeletal muscles of both TA and gastrocnemius between ob/ob and control mice.

### Regeneration of Skeletal Muscles Were Likely to Be Delayed After the Ischemic Injury in Ob/ob Mice, and Administration of Edaravone Was Effective in Such Conditions

**FIGURE 6:**
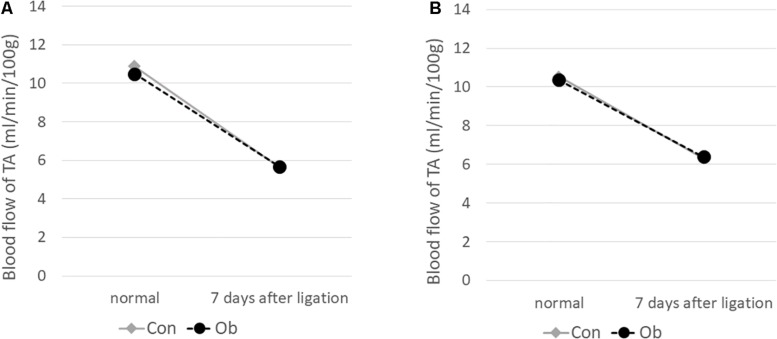
Blood flow decreased after 7 days of ligation of the femoral artery. No obvious difference was seen between control mice (Con) and obese mice (Ob).

**FIGURE 7:**
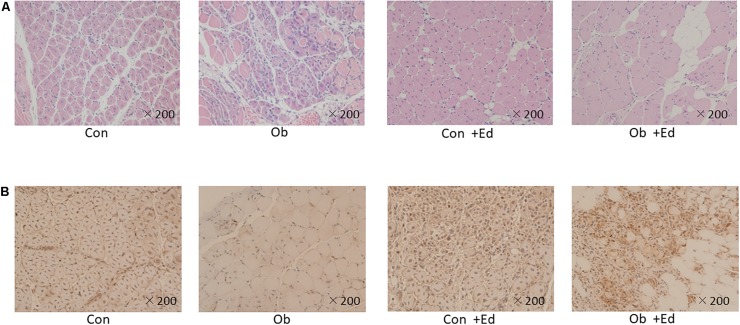
**(A)** HE staining of muscle tissue after artery ligation shows that the cells are round, and reveals infiltration of inflammatory cells in both control (Con) and ob/ob (Ob) mice. **(B)** MyoD immunostaining was lower in Ob than Con at 7 days after ligation of the femoral artery. However, pretreatment with edaravone increases the expression of MyoD in obese mice.

**FIGURE 8:**
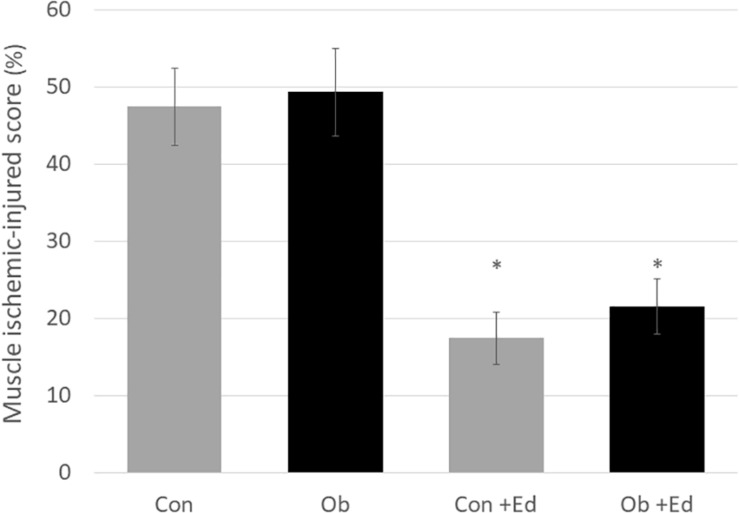
The muscle ischemic-injured score showed that pretreatment with the edaravone significantly inhibited muscle injury at 7 days after ischemia in both control (Con) and obese (Ob) mice (^∗^ compared to mice without administration of edaravone, *p* < 0.05).

**FIGURE 9:**
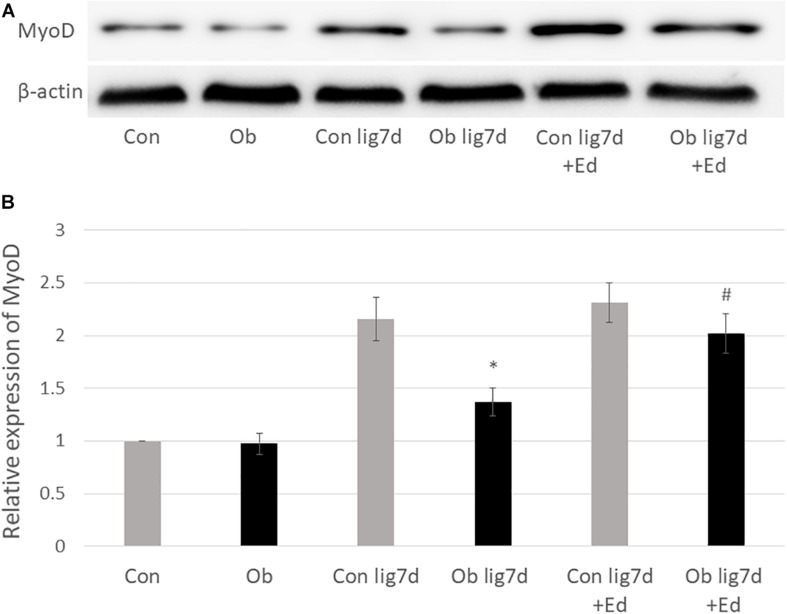
**(A)** Western blotting with anti-MyoD antibody (upper) and β-actin (lower) in control (Con), ob/ob (Ob) mice. **(B)** Band intensities were quantified by ImageJ software and represented as relative expression of MyoD in Con. In Ob, MyoD expression was significantly lower than that in Con at 7 days after arterial ligation (lig 7d), and pretreatment with edaravone (Ed) significantly increased the expression of MyoD in Ob (^∗^ compared with control mice at 7 days after ligation, *p* < 0.01; # compared with Ob at 7 days after ligation, *p* < 0.05).

The ligation of femoral artery remarkably decreased the blood flow of the skeletal muscles in both groups. Nevertheless, necrotic findings of the hind limbs were not macroscopically observed 7 days after the ligation, neither significant differences in blood flow of hind limb muscles were found between ob/ob and control mice (Figure 6). At that time, the histologic examination showed ischemic findings in the skeletal muscles with loss of muscle architecture, as well as inflammatory infiltrate (Figure 7A). The mean value of muscle injury score after the ligation was 47.4 ± 5.1% (527/1112) and 49.3 ± 5.6% (530/1074) in control mice and ob/ob mice, respectively, without statistical differences between both groups. Likewise, pretreatment with edaravone at 3 mg/kg significantly inhibited the injured myofibers by 17.4 ± 3.4% (182/1047) in the control mice and 21.5 ± 4.0% (202/941) in the ob/ob mice at the 7th day (Figure 8). Furthermore, skeletal muscles were weakly immunolabeled for MyoD in ob/ob mice compared to control mice during regeneration, while pretreatment with edaravone upregulated the expression of MyoD in ob/ob mice (Figure 7B). Western blotting also showed that expression of MyoD was significantly decreased in ob/ob mice in comparison with control mice after the ligation of the femoral artery. Nevertheless, pretreatment with edaravone upregulated the expression of MyoD in both control mice and ob/ob mice after the ischemic injury, due to the ligation of the femoral artery, consistent with results of the immunohistochemical analysis (Figure 9).

## Discussion

Although the pathophysiology of the atrophy of the skeletal muscle has not been fully elucidated, oxidative stress could be regarded as a causal factor for this condition. In fact, the marker of oxidative stress was increased in the skeletal muscles and plasma in several diseases including malignant tumors, diabetic mellitus, and obesity (Barreiro et al., 2005; Sainz et al., 2010). Therefore, we firstly tested a direct effect of edaravone, which is clinically used to scavenge free radicals for use in the treatment of cerebral infarction and amyotrophic lateral sclerosis in Japan, in the skeletal muscle cells under oxidative stress mediated by H_2_O_2_. As a result, the present study showed a significantly protective effect to oxidative stress in C2C12 mouse myoblasts in the proliferative assay, similarly to previous studies on inhibitory effects against the oxidative stress on other organs (Yoshida et al., 2006; Hayashi et al., 2014; Hassan et al., 2015).

Furthermore, the previous studies showed that edaravone inhibited the expression of inflammatory cytokines, although this drug did not have an anti-inflammatory effect. The pathophysiology suggested that the edaravone indirectly suppressed inflammation through the inhibition of the cell death mechanism following exposure to oxidative stress. However, several reports have demonstrated that administration of edaravone inhibited systemic or local inflammation associated with oxidative stress in various tissues. It suggested that edaravone indirectly suppresses inflammation by protecting against tissue damage via scavenging of free radicals (Zhang et al., 2005; Yoshida et al., 2006; Fujiwara et al., 2016; Wang et al., 2017). In this study, edaravone showed inhibitory effects against lipid peroxidation and inflammatory cytokines mediated by H_2_O_2_ on C2C12 mouse myoblasts, suggesting that this drug, by scavenging free radicals, could protect skeletal muscle cells exposed to oxidative stress by direct protection of cytoplasm as well as indirect inhibition of inflammation.

The oxidative stress plays a critical role in the pathogenesis of various diseases (Brownlee, 2001). Also, obesity, which causes chronic inflammation of various tissues due to the accumulation of ectopic lipids, including in the skeletal muscles, increases oxidative stress resulting in various organic disorders such as cardiovascular diseases, renal disorders, and hepatic disorders. In fact, Gamez-Mendez et al. (2015) showed increased oxidative stress and arterial stenosis in coronary arteries of obese mice fed a high fat diet. Quigley et al. (2009) reported increased renal oxidative stress and increased urinary albumin excretion in obese mice. In addition, the oxidative stress was shown to be one of the major causative factors for the atrophy of the skeletal muscles due to disuse and malignant tumors (Powers et al., 2007; Fukawa et al., 2016). Therefore, exposition of skeletal muscles to oxidative stress may lead to atrophy and weakness in obesity. The present study showed that ob/ob mice, which were used as one of the most common models for research studies on obesity, had skeletal muscles with a lower wet weight than those in control mice. The skeletal muscles of ob/ob mice had atrophic myofibers without a multiangle shape in the histological analysis and high levels of MDA. It could be considered that the peroxidation of myofibers, due to the increased oxidative stress from accumulation of ectopic lipid, was involved in the atrophy of muscle mass.

Furthermore, we paid attention to the disturbance in the blood flow in the atrophy of the skeletal muscles in obesity, because it is well-known its association with cardiovascular diseases. In fact, atherosclerosis was frequently observed in obese individuals. In addition, ob/ob mice had pathological findings in cardiovascular tissues, including premature cardiac dysfunction, and reduced diameter of aorta and cardiac arteries (Di Lascio et al., 2018). Nevertheless, there was no change of blood flow in skeletal muscles between ob/ob and control mice. It suggested that obesity alone did not represent a harmful effect to blood flow in peripheral arteries vascularizing the skeletal muscles.

Repeatedly, obesity is a risk factor for many vascular diseases, including peripheral artery disease of the lower extremities, as well as cardiovascular diseases (Marseglia et al., 2014; Manna and Jain, 2015), especially in elderly patients. Therefore, we estimated that skeletal muscle blood flow is decreased in obese mice, even though the decrease of blood flow could not be found in the obesity alone. However, the present study showed that the expression of MyoD was lower in the ob/ob mice than in the control mice following the ligation of the femoral artery. This finding suggested that obese individuals had an inferior ability to regenerate the skeletal muscle after an ischemic injury. Nguyen et al. (2011) also reported delayed muscle regeneration in ob/ob mice with muscular damage caused by injecting cardiotoxin into skeletal muscle. These results, including ours, are considered to be important regarding the pathophysiology of sarcopenic obesity, since there are many middle and high aged patients with peripheral arterial disease in conjunction with metabolic syndrome, represented by obesity. It is inferred that muscle atrophy might be accelerated by the impairment of muscle regeneration in obesity, when the blood flow is decreased due to peripheral arterial diseases, which is considered to be part of the pathophysiology in sarcopenic obesity.

Therefore, edaravone might be useful for the atrophy of skeletal muscles due to deterioration of blood flow in conjunction with obesity, since previous studies had shown the protective effect of this drug in various tissues after ischemic injury (Hori et al., 2013; Kikuchi et al., 2013; Ni et al., 2018). In fact, the present study also showed that edaravone administration significantly upregulated the expression of MyoD in ob/ob mice after ligation of the femoral artery, indicating the activation of myoblasts differentiated from myosatellite cells and their fusion into mature myofibers in the regeneration of muscle tissues. Besides, edaravone protected C2C12 mouse myoblasts from the oxidative stress through the inhibition of lipid peroxidation and inflammatory cytokines. Therefore, we suggest that edaravone could protect the skeletal muscles via reduction of oxidative stress in arteries under conditions of obesity. Nonetheless, there existed major limitations to this study. The other regulator should have been examined in the regeneration of the skeletal muscles. In addition, the ability of myosatellite cells to proliferate and differentiate under obesity conditions should have been evaluated.

## Conclusion

The administration of edaravone effectively protected the C2C12 mouse myoblasts exposed to oxidative stress by using H_2_O_2_, with a significant inhibition of TNF-α expression. In ob/ob mice, the skeletal muscles had significantly more atrophic-myofibers with a high level of lipid peroxidation. Furthermore, blood flow to the skeletal muscles in ob/ob mice was not different from that in the control mice, while the expression of MyoD was decreased during the regeneration of skeletal muscles after ischemic injury in ob/ob mice. These findings suggested that obesity alone did not represent a harmful effect to skeletal muscles, but their regeneration could be impaired after the ischemic injury in ob/ob mice. Edaravone could be useful for regeneration after ischemic injury in ob/ob mice. We suggest that attenuation of the regenerative ability due to ischemic injury could have a causal role in the atrophy of skeletal muscles in obese mice, as a result of peripheral arterial diseases related to obesity.

## Data Availability Statement

The datasets used and/or analyzed during the current study are available from the corresponding author on reasonable request.

## Ethics Statement

The experimental protocol was approved by the committee of animal research at Mie University.

## Author Contributions

TN and MT designed the research and wrote the manuscript. TI and TA participated in the experimental design and techniques. AS did final editing. All authors read and approved the final manuscript.

## Conflict of Interest

The authors declare that the research was conducted in the absence of any commercial or financial relationships that could be construed as a potential conflict of interest.

## Abbreviations

BSA: bovine serum albumin
cDNA: complementary deoxyribo nucleic acid
DAB: 3,3 ′ -diaminobenzidine tetrahydrochloride
DMEM: Dulbecco’s modified Eagle’s medium
Ed: edaravone
EDTA: ethylene diamino tetraacetic acid
FBS: fetal bovine serum
GC: gastrocnemius
GAPDH: glyceraldehyde-3-phosphate dehydrogenase
HE: hematoxylin and eosin
HRP: horseradish peroxidase
MDA: malondialdehyde
MTS: 3-(4,5-dimethylthiazol-2-yl)-5-(3-carboxymethoxyphenyl)-2-(4-sulfophenyl)-2H-tetrazolium
PCR: polymerase chain reaction
RIPA: radioimmunoprecipitation assay
RNA: ribo nucleic acid
ROS: reactive oxygen species
SDS: sodium dodecyl sulfate
SDS-PAGE: sodium dodecyl sulfate polyacrylamide gel electrophoresis
TA: tibialis anterior
TBA: thiobarbituric acid
TBARS: thiobarbituric acid reactive substances
TBS: tris-buffered saline
TNF-α: tumor necrosis factor alpha.
